# Reply to Shah, J.S.; Ramasamy, R. Target Antigens in Western and Line Immunoblots for Supporting the Diagnosis of Lyme Disease. Comment on “Porwancher et al. Immunoblot Criteria for Diagnosis of Lyme Disease: A Comparison of CDC Criteria to Alternative Interpretive Approaches. *Pathogens* 2023, *12*, 1282”

**DOI:** 10.3390/pathogens13050353

**Published:** 2024-04-25

**Authors:** Richard Porwancher, Andrew Levin, Rosalie Trevejo

**Affiliations:** 1 Section of Allergy, Immunology, and Infectious Diseases, Department of Medicine, Rutgers Robert Wood Johnson Medical School, New Brunswick, NJ 08901, USA; 2 Princeton Infectious Diseases Associates, LLC, Plainsboro, NJ 08536, USA; 3 Kephera Diagnostics, LLC, Framingham, MA 01702, USA; alevin@kephera.com; 4 Acute and Communicable Disease Prevention, Oregon Health Authority, Portland, OR 97232, USA; rosalie.trevejo2@oha.oregon.gov

We are writing in response to comments made by Shah and Ramasamy [1] concerning our recently published article entitled, “Immunoblot Criteria for Diagnosis of Lyme Disease: A Comparison of CDC Criteria to Alternative Interpretive Approaches” [2]. The purpose of our article was to compare the performance of two commercially utilized alternative interpretive criteria for immunoblots to CDC interpretive criteria for diagnosis of Lyme disease (LD) in the United States. We did not directly evaluate the in vitro performance of these laboratory-developed immunoblots or their application for diagnosis of European LD; instead, we evaluated the interpretive criteria utilized by these laboratory-developed immunoblots for diagnosis of US Lyme disease by applying them to two FDA-cleared Western blot test kits and one FDA-cleared line blot test kit. We recognize that the in vitro performance of laboratory-developed immunoblots might differ from FDA-cleared immunoblots and we acknowledged that limitation in our article. There is little published information concerning the performance of these laboratory developed immunoblots, so we also reviewed the available literature, focusing on study design and the claims made by these laboratories concerning their performance relative to CDC-advocated techniques [3,4,5,6]. Alternative Criteria A, listed in Table 1 of our article [2], were adapted from publications from IGeneX, Inc. (Milipitas, CA, USA), as well as information provided on their website [3,4,5,7,8]. We also compared the performance of an FDA-cleared modified 2-tiered approach to IGeneX alternative line blot criteria for serodiagnosis of US Lyme disease using data from the CDC Lyme Serum Repository (LSR) (dataset 8) [2,9]. 

One of the principal features of the immunoblots utilized by IGeneX is the use of multiple strains or genospecies of *Borrelia burgdorferi* sensu lato for serodiagnosis. Shah et al. [3,4] discuss the use of two strains of *B. burgdorferi* sensu stricto for Western blotting. Shah and Ramasamy [1] correctly point out that the pilot study by Lui et al. [5] describing a multi-strain recombinant line blot did not specify the exact number of strains or genospecies utilized, referring only to “recombinant proteins derived from several US and European species of BBsl [*Borrelia burgdorferi* sensu lato]…”. The IGeneX website [7,8], however, reported using eight different *Borrelia burgdorferi* sensu lato strains or genospecies in their line blot prior to publication of our article [2] and currently employs nine different *Borrelia burgdorferi* sensu lato strains or genospecies [7]. The IGeneX website [7], as well as Shah and Ramasamy [1], refer to the article by Liu et al. [5] as a reference for the performance of the IGeneX recombinant line blot. Our key concern is not the exact number of strains/genospecies included in IGeneX’s recombinant line blot but the scientific justification for its decision to utilize multiple strains/genospecies for diagnosis of US LD. Apart from a few isolated case reports, there is no microbiologic data (i.e., culture or DNA evidence) that demonstrates a significant role for genospecies other than *B. burgdorferi* sensu stricto as a cause of human LD in the US [10]. The specificity of serologic methods used to claim endemic LD in Mexico has been questioned [10] and the occasional isolation of *Borrelia garinii* from seabirds in Labrador and mice in South Carolina do not appear to have human correlates [11]. Even *Borrelia mayonii*, the most recently recognized US species of *B. burgdorferi* sensu lato, is found only rarely in the upper Midwest [12]. No data were presented by Shah and Ramasamy [1] to examine the trade-off between immunoblot sensitivity and specificity by utilizing more than one *B. burgdorferi* strain or genospecies. Shah and Ramasamy [1] misstate our concerns about reproducibility; we acknowledge that IGeneX uses a weak-positive control band for immunoblot interpretation, but our article [2] correctly states that “justification of weak-positive band density and immunoblot reproducibility studies were not reported by either Shah [3,4] or Liu [5]”. (See page 5, paragraph 1 of our article [2]).

Similarly, no data were provided by Shah and Ramasamy to determine the trade-off between sensitivity and specificity by including antibodies to OspA and OspB in the IGeneX recombinant line blot panel (i.e., 31 kDa and 34 kDa bands). Prior literature suggests that the 31 kDa and 34 kDa bands provided no significant benefit for detection of early LD using Western blots [13]. In one small study of partially treated patients with late-stage LD, adding the 31 kDa and 34 kDa bands to the CDC-advocated Western blot panel demonstrated 8% greater sensitivity [14]. Even without utilizing the 31 kDa and 34 kDa bands, line blot data from the CDC LSR demonstrated that IGeneX alternative immunoblot criteria already detected 100% of late-stage cases but at a cost of 17.7% false-positives in healthy controls (see text and Table 12) [2]. The use of recombinant antigens in line blots is not a guarantee of specificity; as discussed in our article [2], multiple infectious and non-infectious diseases can lead to cross-reactions [15,16]. 

The Shah [3,4] and Liu [5] studies report only single-tier performance of their respective multi-strain immunoblots and utilize both IgG and IgM antibodies for LD serodiagnosis irrespective of disease duration. Liu et al. [5] compared the sensitivity and specificity of their recombinant line blot to a two-tiered approach that utilized a multi-strain Western blot for 127 out of 152 control sera, claiming that the “… the specificity of the Lyme IB [recombinant line blot] is equivalent to two-tiered testing (using whole-cell lysate EIA and Western blot) with improved sensitivity (Table 5)”. Shah and Ramasamy acknowledge that the Liu article [5] is the only publication that addresses the performance of their recombinant line blot relative to CDC criteria. The IGeneX website [7,8] claims that their recombinant line blot “… is more sensitive and specific than the ELISA, IFA, and traditional Western Blot tests for Lyme”. The IGeneX website [7] also states that their recombinant line blot “detects the full spectrum of disease: early, active, and late-stage” and “does not require a confirmation test”. Shah and Ramasamy [1] noted that a positive or equivocal LD screening assay (LSA) should be confirmed by a second-tier assay such as IgG and IgM immunoblots, as recommended by the CDC [17,18]. In contrast to CDC criteria [17,18], IGeneX immunoblot criteria [7,8] do not recommend a positive or equivocal first-tier screening assay *before* performing either a Western blot or line blot. The IGeneX website [8] mentions using IgM immunoblots for diagnosis of “early disease/re-activation later” but does not define the term “re-activation”. Although IGeneX recommends performing IgM line blots for LD diagnosis “at least two weeks after possible exposure”, it does not limit the use of IgM immunoblots for LD diagnosis to the first 30 days of illness [8]. In contrast, CDC guidance [17,18] recommends utilizing only IgG immunoblots for LD serodiagnosis more than 30 days after disease onset to reduce the risk of false-positive IgM serology. The Liu study [5], however, utilized both IgG and IgM recombinant line blots for diagnosis of all stages of LD. Although Shah and Ramasamy [1] characterize the articles by Shah [3,4] and Liu [5] as exploratory, it is clear from the interpretive criteria published on the IGeneX website [7,8] that IGeneX permits using multi-strain Western blots and line blots as single-tier assays for LD serodiagnosis; neither a first-tier screening assay nor confirmatory assay is recommended by IGeneX before reporting immunoblot results positive by alternative criteria. We believe that we are justified in evaluating the performance of the IGeneX IgG and IgM alternative immunoblot criteria as a single-tier approach, irrespective of disease duration. 

We remain concerned about the study design used by IGeneX to support their claim of equivalent specificity to CDC-advocated methods. The Shah [3,4] and Lui [5] studies utilized controls from proficiency test samples and sera prescreened for antibodies to *B. burgdorferi.* The CDC advocates using control samples from individual patients to determine assay specificity [17,18]; proficiency test sera may, however, utilize pooled or duplicate samples. By utilizing prescreened sera as controls, the specificity of the immunoblots studied by Shah [3,4] and Liu [5] might have been artificially increased by eliminating sera with cross-reacting antibodies. The recombinant line blot studied by Liu [5] was largely compared to a laboratory-developed multi-strain Western blot, a methodology that is not advocated by the CDC [17,18]. Finally, line blot reproducibility studies were not reported in the Liu study [5]. Absent other supporting data, we believe that it is premature to claim that the IGeneX recombinant line blot is as specific as the CDC-advocated two-tiered approach. 

Shah and Ramasamy [1] state that physicians who order an IGeneX immunoblot always receive two interpretations, one using IGeneX alternative interpretive criteria and another using “CDC criteria”. Utilizing CDC guidance to interpret immunoblot band results is not the same, however, as providing an assay consistent with CDC standards [17,18]. Because the composition of the IGeneX multi-strain immunoblots and their standardization differ significantly from CDC-advocated methods [17,18], it is not clear that CDC interpretive criteria can be applied to the IGeneX immunoblots. Even if we assume that the IGeneX line blot provides performance equivalent to FDA-cleared line blots, IGeneX does not consistently apply the CDC-advocated two-tiered paradigm to its line blots: (i) first-tier assays are not recommended before performing IGeneX line blots, and (ii) IGeneX permits utilizing IgM line blots for serodiagnosis in patients whose duration of illness exceeds 30 days. When reporting IGeneX line blot results using “CDC criteria” it is important that physicians are informed about both technical and interpretive differences between CDC-advocated standards and IGeneX line blots that might influence test interpretation. 

Shah and Ramasamy [1] misstate the composition and reporting of our datasets; except for Dataset 8 (CDC Lyme Serum Repository (LSR)), all individual patient data available to us were reported in full in our article and Supplementary Materials. Individual patient data were available for only 3 datasets (Datasets 1, 4, and 8). Individual patient data used to examine the performance of IGeneX alternative immunoblot criteria for IgG antibody in healthy controls were reported in full for Datasets 1 and 4 (Supplementary Files S3 and S4). Individual patient data utilizing IgG line blot data from the CDC LSR (Dataset 8) were analyzed by us and the performance of both IGeneX and CDC immunoblot criteria were reported in summary fashion in our article [2]. Individual patient IgM immunoblot data were also available from Datasets 4 and 8 and reported either in full (Dataset 4) or in summary fashion (dataset 8). The frequency of antibody responses to individual immunoblot bands was reported in healthy controls from all datasets (1–8), including the 31 kDa and 34 kDa bands from Datasets 1 and 4 (Supplementary Tables S1 and S2). Of 159 patient specimens processed by Trevejo et al. [13], individual immunoblot results were available for 158 patient specimens and are reported in full in Supplementary File S4 (Dataset 4) [2]; one immunoblot result was missing because it was never performed due to insufficient quantity of serum, not because of selective reporting on our part. Dataset 4 did not include controls with potentially cross-reacting medical conditions because they were not part of the original study design [13]. All data obtained solely from FDA sources were reported in summary fashion because individual patient data were unavailable.

The reason for summary reporting from the CDC LSR is to preserve its value for standardization of Lyme disease assays. The CDC LSR is a unique public resource available to device manufacturers and researchers for development of new LD assays [19]. Sera from LD patients and controls are released in stages to laboratory test developers, including a final validation set [13]. Test developers are blinded to sample category (i.e., LD patient or control) to provide an objective assessment of investigational test performance. Under a materials transfer agreement, one of our study investigators (R.P.) was provided with partially blinded data from the LSR from studies previously conducted by the CDC. We did not receive any serum from the LSR, only pre-existing data. In order to preserve the value of the LSR for future use, we were not allowed to publish data from individual serum samples; only summary data were published with the CDC’s permission. Shah and Ramasamy’s assumption that the data we obtained from the LSR is unavailable to other researchers is inaccurate; interested parties can contact the CDC Division of Vector-Borne Diseases in Fort Collins, Colorado, USA to discuss dataset access. 

We acknowledged that our datasets were incomplete [2], but this missing data did not affect the validity of our key finding, the high false-positive rates in healthy controls in immunoblots interpreted using single-tier alternative criteria. Although the LSR (Dataset 8) did not record results to the 31 kDa and 34 kDa immunoblot bands, this dataset was still sufficient to provide substantive information about the specificity of IGeneX interpretive criteria when applied to Viramed ViraStripe IgG and IgM line blots (Table 12) [2]. We identified false-positive rates (FPRs) of 17.7% in healthy controls and 19.4% in individuals with potentially cross-reacting medical conditions when applying single-tier IGeneX IgG and IgM line blot criteria [8] to the full LSR dataset [2,19]. Had 31 kDa and 34 kDa band results been available, there would have been even greater opportunity to generate false-positive band combinations in the LSR control samples (Dataset 8). The latter phenomenon was observed when determining the FPR of single-tier IgG Western blots in Dataset 1 that were positive by IGeneX interpretive criteria (as reported in Tables 4 and 5); the FPR observed for single-tier IgG Western blots positive by IGeneX interpretive criteria increased from 24.9% to 29.8% in healthy controls after including the 31 kDa and 34 kDa bands [2]. Because sera are typically tested using both IgG and IgM immunoblots, single-tier IgM immunoblot results from Dataset 1 could only have added to the already high FPR (29.8%) observed for single-tier IgG Western blots interpreted using IGeneX alternative criteria [8]. Had individuals with potentially cross-reacting medical conditions been included in Datasets 1 and 4, they too may have increased the overall FPRs. Because the datasets are incomplete, the reported FPRs in healthy controls for Datasets 1, 4, and 8 represent conservative estimates of the true FPRs for single-tier alternative immunoblot criteria. 

We noted variability in sensitivity, specificity, and band frequency among our datasets (i.e., dataset heterogeneity). Although Shah and Ramasamy [1] claim we used “uncommonly complex statistical analyses”, a random effect meta-analysis is, in fact, the most commonly used statistical method to analyze heterogeneous datasets [20]. This type of meta-analysis weights the contribution of each dataset to provide the least biased estimate of interpretive criteria performance. A PubMed search using the title term “meta-analysis” yielded over 186,000 articles [21], confirming its ubiquity. Utilizing the three datasets with individual patient data (1, 4, and 8), but without including the 31 kDa and 34 kDa bands, a random effect meta-analysis in Table 5 reports the FPR of single-tier IGeneX IgG immunoblot criteria as 12.9% in healthy controls; in contrast, the FPR for single-tier IgG immunoblots using CDC criteria was 2.4%, a statistically significant difference (*p* < 0.05) [2]. Also in Table 5, a meta-analysis of single-tier IgM immunoblots using IGeneX and CDC interpretive criteria applied to Datasets 4 and 8 demonstrated the same FPR in healthy controls (i.e. 7.1%) [2]; the 31 kDa and 34 kDa bands were not utilized for IgM immunoblots from these datasets, potentially aiding the specificity of IGeneX alternative criteria. Recognizing that IgG and IgM immunoblots are usually performed together on a given sample, Tables 9 and 10 report the combined performance of IgG and IgM immunoblots interpreted using single-tier IGeneX alternative criteria and CDC 2-tiered criteria, but without including the 31 kDa and 34 kDa bands [2]. Utilizing a random effect meta-analysis, the overall FPR for single-tier IGeneX alternative immunoblot criteria was 12.4% in healthy controls, representing the minimum FPR for those criteria (Table 10). In contrast, we observed a 1.0% overall FPR in healthy controls for two-tiered CDC criteria, as reported in the meta-analysis from Table 9. The 95% confidence intervals of the overall FPRs in healthy controls reported for two-tiered CDC immunoblot criteria and single-tier IGeneX immunoblot criteria reported in Tables 9 and 10, respectively, do not overlap [2], arguing in favor of a significant difference in specificity. 

The study by Sfeir et al. [22], is the most comprehensive evaluation to-date of an FDA-cleared modified two-tiered assay utilizing sera from the CDC LSR [19]; the Sfeir data, reported in Table 12 of our article [2], is a subset of the full LSR because of the protocol used by the CDC to validate new assays [19]. Because we did not have access to individual immunoblot results from this validation subset, we were unable to calculate the performance of alternative immunoblot criteria in this specific subset. The Sfeir data [22] were therefore used to compare the performance of modified two-tiered CDC criteria in this validation subset (last row of Table 12) to IGeneX alternative interpretive criteria applied to line blot data from the entire LSR (first row of Table 12); this modified two-tiered approach demonstrated statistically superior specificity compared to IGeneX alternative interpretive criteria. 

One co-author, R.T., reports no financial conflicts of interest. A second co-author (R.P.) recently donated his patents for LD assays to a non-profit organization and now reports no financial interest in laboratory tests for LD. Our third co-author (A.L.) reports no change in potential conflicts of interest [2]. We note that both Shah and Ramasamy are IGeneX employees [1]. 

We recognize that recombinant line blots utilizing multiple genospecies hold potential to improve immunoblot sensitivity and specificity for European LD, as demonstrated by Goettner et al. [23]. The meta-analyses performed in our study provide the least biased estimates of alternative immunoblot interpretive criteria performance in our US-based datasets. In summary, we observed: (i) incorporating the 31 kDa and 34 kDa bands in the immunoblot panel did not significantly improve immunoblot sensitivity in our datasets, and (ii) standard two-tiered CDC criteria using immunoblots and modified two-tiered CDC criteria were significantly more specific than single-tier IGeneX alternative immunoblot criteria [2]. 
